# Comparative Study of UMCM-9 Polymorphs: Structural, Dynamic, and Hydrogen Storage Properties via Atomistic Simulations

**DOI:** 10.1021/acs.jpcc.4c07872

**Published:** 2025-03-04

**Authors:** Josef M. Gallmetzer, Jakob Gamper, Stefanie Kröll, Thomas S. Hofer

**Affiliations:** Institute of General, Inorganic and Theoretical Chemistry, University of Innsbruck, Innrain 80-82, 6020 Innsbruck, Austria

## Abstract

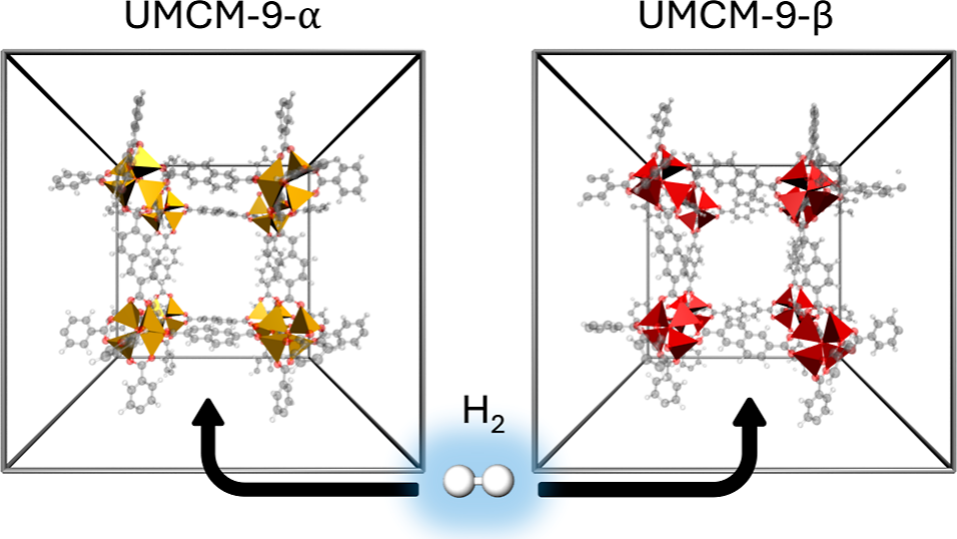

The structural and dynamic properties of two polymorphs of the metal–organic framework UMCM-9 (UMCM-9-α and -β) have been studied via molecular dynamics (MD) simulations in conjunction with density functional tight binding (DFTB) as well as the newly developed MACE–MP neural network potential (NNP). Based on these calculations, a novel UMCM-9-β polymorph is proposed that exhibits reduced linker strain and increased flexibility compared to UMCM-9-α, which is shown to be energetically less stable. UMCM-9-β exhibits enhanced diffusion of molecular hydrogen due to weaker host–guest interactions, whereas UMCM-9-α exhibits stronger interactions, leading to improved hydrogen adsorption. The results suggest that synthesis conditions may control the formation of both polymorphs: UMCM-9-β is likely to be the thermodynamic product, forming under stable conditions, while UMCM-9-α may be the kinetic product, forming under accelerated synthesis conditions. This study highlights the potential for optimizing MOFs for specific gas storage applications to achieve the desired structural and associated gas storage properties.

## 1 Introduction

As the global imperative to decarbonize the economy intensifies, the necessity for pioneering energy storage and delivery systems has become increasingly evident.^1^ Among the various candidates for green energy carriers, hydrogen has attracted considerable attention for its potential to support a low-carbon energy infrastructure.^2,3^ However, the widespread adoption of hydrogen is hindered by significant challenges associated with its storage and transport, as well as its low energy density per unit volume and the difficulties associated with maintaining it in a stable, useable form.^4,5^

Metal–organic frameworks (MOF) have emerged as a promising solution to these challenges.^4,6−8^ MOFs are a class of crystalline, porous materials built of inorganic metal ions or clusters coordinated by organic ligands to form highly tunable structures.^9^ This allows the design of MOFs with specific properties that are useful for gas storage, such as high surface area, tunable pore size and the ability to incorporate functional groups that enhance adsorption.^10^ These enable MOFs to overcome the limitations of traditional gas storage methods and offer the potential for more efficient and scalable storage solutions.^10^ One of the most promising MOF materials for hydrogen storage is UMCM-9 (University of Michigan Crystalline Material-9), which has been shown to exhibit exceptional hydrogen storage capabilities.^11,12^ UMCM-9 is a porous metal–organic framework composed of Zn_4_O^6+^ clusters serving as secondary building units (SBU), connected by equal proportions of naphthalene-2,6-dicarboxylate (NDC) and biphenyl-4,4′-dicarboxylate (BPDC) linkers.^11^ UMCM-9 exhibits a high surface area exceeding 4000 m^2^ g^–1^ and a large pore volume over 1.80 cm^3^ g^–1^, making it an attractive candidate for gas storage applications.^11^ Ahmed et al.^12^ showed that UMCM-9 has a high hydrogen total gravimetric capacity of 11.3 wt % between 100 bar-77 K and 5 bar-160 K.

Supramolecular isomerism remains an underexplored area in the study and analysis of Zn_4_O-based MOFs. For instance, Amirjalayer and Schmid^13^ has highlighted the importance of the SBU orientation in determining the structural properties of isoreticular MOFs, showing that the orientation of the SBUs can significantly impact the framework topology. Understanding such behavior in Zn_4_O-based systems provides a valuable foundation for interpreting polymorphism in UMCM-9, where the distinct SBU orientations result in unique gas storage and diffusion characteristics.

Despite progress in the development of MOFs for gas storage, there is still a need for an in-depth understanding of the fundamental mechanisms that govern gas adsorption and storage in these materials,^12^ particularly for UMCM-9. While experimental studies^11,12^ have provided valuable insights, they are often limited by the inability to directly observe the interactions and dynamics of gases within the MOF pores.

In recent decades, theoretical modeling has advanced substantially, providing robust methods to simulate and understand chemical systems at an atomic level. Empirical potential models, such as molecular mechanics (MM) or force fields (FF), have proven highly effective in characterizing MOF systems.^14,15^ However, these methods often struggle with accurately representing intricate phenomena, including polarization, charge transfer, and many-body interactions. These challenges become particularly relevant when addressing the behavior of open metal sites^10^ or the interactions of guest molecules within MOFs,^16−18^ emphasizing the need for methodologies that balance computational efficiency with high accuracy.

Atomistic simulations, such as molecular dynamics (MD), combined with density functional tight binding^19,20^ (DFTB) calculations provide a versatile tool for investigating the behavior of gases in MOFs due to their high accuracy and efficient computation, compared to less rigorous MM–MD and expensive QM–MD.^21−25^ These simulations allow the exploration of gas adsorption sites, diffusion pathways and the impact of various structural modifications on storage capacity and stability.^26−28^

Additionally, new advances in machine learning-based neural network potentials (NNPs) have enabled the accurate description of complex interactions in nanoporous compounds with even higher computational efficiency while maintaining reasonable chemical accuracy.^29−34^ These innovations have opened up new possibilities for studying the behavior of gases in MOF compounds, since larger time scales can be achieved required for the study of diffusion phenomena.^21,35^ By providing a detailed molecular-level understanding of these processes, simulations can guide the design of new MOFs with optimized properties for gas storage, potentially leading to breakthroughs that experimental methods alone might miss.

This work aims to address the current gaps in the literature by advanced molecular simulations to investigate structural modification of the SBUs and hydrogen storage capabilities of UMCM-9.^11,12^ This integrated approach aims to provide a comprehensive understanding of how hydrogen interacts with UMCM-9 at the atomic level. Furthermore, two polymorphs, UMCM-9-α and -β, have been studied to investigate the impact of structural modifications on the hydrogen storage properties of the material.

## 2 Computational Details

Molecular dynamics (MD) simulations have been conducted using the PQ(36) (version 0.5.0) software package, interfaced with the DFTB+ program^37^ for density functional tight binding (DFTB) calculations and with MACE(38,39) via the ASE(40) package for neural network potential (NNP) calculations to describe energies and forces.

The DFTB calculations have employed the third-order self-consistent charge density-functional tight-binding (DFTB3) method using the 3ob parameter set.^41,42^ Additionally, DFT-D3 dispersion corrections with Becke-Johnson damping^43,44^ of the associated parameter set have been applied, including hydrogen damping with an exponential factor of 4.0.^45,46^

Furthermore, the MACE interaction potential^38,39^ has been employed as a neural network potential (NNP) to describe the system. Specifically, the MACE–MP model (version 0, medium model size and floating point–accuracy as recommended for MD)^34^ has been used in conjunction with DFT-D3 dispersion corrections and Becke-Johnson damping.^44^

To sample the *NVT* and *NPT* ensembles, the Nosé–Hoover thermostat^47−49^ with a chain length of 5 and a coupling frequency of 1000 cm^–1^ has been used for temperature control, while the pressure has been controlled using the Berendsen barostat^50^ with a pressure relaxation time τ_p_ = 1.0 ps. All *NPT* simulations have been performed at 1 atm. For time integration of the system, the velocity-Verlet algorithm^51^ has been used. All covalent bonds involving hydrogen atoms have been constrained using the SHAKE/RATTLE algorithm^52,53^ to enable the use of a larger time step of 2.0 fs. The equilibrium bond lengths used for the constraints have been determined from *NPT*-equilibrated MD trajectories run with a time step of 0.5 fs for a minimum of 25 ps.

A more detailed description of the simulation protocol can be found in the Supporting Information. The simulations have been analyzed as described in detail in a previous work.^21^ The analysis has been in part performed using the PQAnalysis package.^54^ PXRD patterns have been generated using RIETAN–FP^55^ as implemented in the VESTA program.^56^

In addition, DFT structure optimizations have been performed using Gaussian 16(57) at the PBE level of theory^58,59^ with the 6-31G^60,61^ basis set for C, H, and O atoms, and the LANL08^62,63^ effective core potential (LANL2DZ ECP) for Zn atoms. Additionally, the D3 dispersion correction of Grimme with Becke-Johnson damping^44^ has been applied. The basis sets have been taken from the Basis Set Exchange^64−66^ database. The DFT calculations have been applied to a nonperiodic model system comprised of two Zn_4_O^6+^ clusters capped with formate groups and connected by an NDC linker referred to as 2SBU-NDC, analogous to the system employed by Amirjalayer and Schmid.^13^ In order to model the conformation present in the UMCM-9-α crystal structure, the torsion angles of the NDC linker have been constrained.

## 3 Structural Details

**Figure 1:**
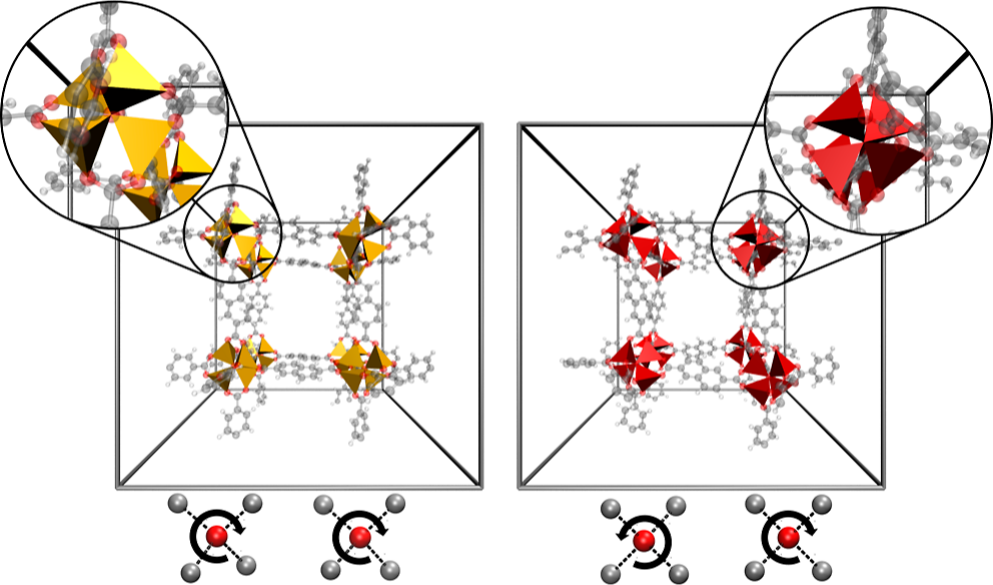
(Left) the structure of UMCM-9-α as reported by Koh et al.^11^ (right). The proposed structure of UMCM-9-β with a modified orientation of the Zn_4_O^6+^ clusters.

The cubic unit cell of UMCM-9 contains 8 Zn_4_O^6+^ clusters as well as 12 NDC linkers and 12 BPDC linkers, with a lattice parameter of 32.5 Å and crystallizes in the space group *P*23 (no. 195). UMCM-9 is based on the same Zn_4_O^6+^ cluster as also found in MOF-5. However, the published crystal structure^11^ differs in the orientation of these inorganic nodes. In MOF-5 the clusters are oriented symmetric with respect to a mirror plane, i.e. alternating orientation, while in UMCM-9 the cluster are translational copies, see Figure 1.

Due to the high strain observed in the carboxylate groups of the linkers in the original structure proposed by Koh et al.,^11^ referred to as UMCM-9-α in this work, a novel conformational polymorphic structure UMCM-9-β is proposed. The strain is especially pronounced in the carboxylate groups of the NDC linkers, which are oriented in a nonideal configuration in the original structure due to the orientation of the Zn_4_O^6+^ clusters, as illustrated in Figure 3. This new structure has been constructed by mirroring half of the SBUs along each principal axis, similar to the SBU orientations found in MOF-5.^67^ The proposed configuration aims to alleviate the strain in the SBUs and linkers present in the original model structure.

## 4 Results and Discussion

### 4.1 Energetic Analysis

The difference in potential energy Δ*E* of UMCM-9-α and -β has been calculated *via* energy minimization of the structures using DFTB3 and MACE–MP, as well as from the *NPT* MD simulations at 298.15 K and 1 atm. Additionally, Δ*E* has also been determined from DFT calculations of the 2SBU-NDC model system.

From Table S4 it can be seen that the strain of UMCM-9-α has a considerable impact on the potential energy of the system, with a significantly higher potential energy compared to the β-form. The difference in total energy obtained via energy minimization is 106.1 and 77.2 kJ mol^–1^ for DFTB3 and MACE–MP, showing the proposed structure UMCM-9-β is energetically more favorable. When considering the average potential energy derived from the *NPT* simulations, the energy difference between the two polymorphs is found to be 125.9 and 130.9 kJ mol^–1^ for DFTB3 and MACE–MP, respectively. The DFT calculations show that when the UMCM9-α model system is optimized without any constraints applied, the energy difference between both forms is found to be only 0.3 J mol^–1^ higher, which is negligible. However, when constraining the NDC linker’s torsion angles, the energy of the α-form has been found to be 15.5 kJ mol^–1^ higher than that of the unconstrained β-model.

Due to its lower energy, UMCM-9-β could be considered to be the thermodynamically more stable product, while the α-form could potentially be formed as a kinetic product. Therefore, the application of different synthesis conditions for UMCM-9 has the potential to control the formation of these polymorphs. Different synthesis conditions,^68−70^ solvent and/or temperature, could be used to steer the formation of these two polymorphs.

### 4.2 Structural Validation

As can be observed in Table S5, the lattice parameter *a* of UMCM-9-α and -β has been calculated via energy minimization and MD simulations using DFTB3 and MACE–MP, respectively. The lattice parameters derived from the geometry optimization of the α-form are found to be 32.5 and 32.3 Å for DFTB3 and MACE–MP, while those of the β-form are 32.6 and 32.4 Å, respectively. When comparing the lattice parameters derived from a simulation at 298.15 K and 1 atm, the average lattice parameters of the α-form are found to be 32.5 and 32.1 Å for DFTB3 and MACE–MP, respectively. Whereas, UMCM-9-β has an average *a* of 32.5 Å for DFTB3 and 32.1 Å for MACE–MP.

In general, UMCM-9-α is found to be in closer agreement with the experimental value of 32.5 Å, reported by Koh et al.,^11^ than those of UMCM-9-β when DFTB3 is employed. The lattice parameter of UMCM-9-β, as calculated using MACE–MP, are found to be in closer agreement with the experimental values than those of UMCM-9-α. With an error of less than 0.2 Å of the experimental values, which corresponds to 0.6% of the total lattice parameter, both methods are capable of predicting the lattice parameters of UMCM-9-α and -β with good accuracy. In the case of the average lattice parameters derived from the MD simulations, the DFTB3 simulations yield a closer agreement for both systems in comparison to the MACE–MP simulations, which show a larger deviation from the experimental values.

It should be noted that the optimization of molecular structures corresponds to a 0 K energy minimum, which does not represent nonzero thermal experimental conditions. Therefore, the lattice parameters derived from the MD simulations at 298.15 K and 1 atm are more representative when compared to the experimental reference.

**Figure 2:**
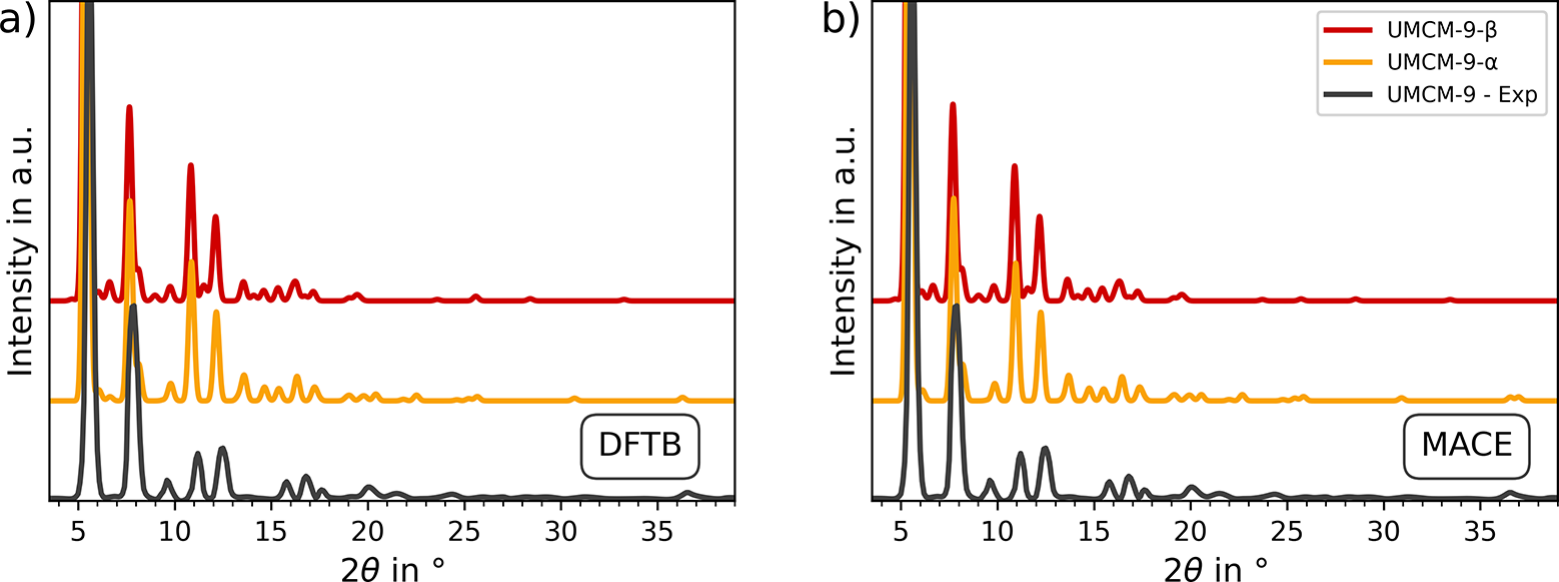
PXRD patterns (λ_Cu K_α__ = 1.5406 Å) of the α- and β-form of UMCM-9 obtained from MD simulations at 298.15 K and 1 atm using (a) DFTB3 and (b) MACE–MP, respectively. The reference experimental patterns was taken from Koh et al.^11^ and is shown in black.

In order to investigate whether crystallographic measurements could potentially discriminate between the two polymorphs, ensemble-averaged powder X-ray diffraction (PXRD) patterns of UMCM-9-α and -β have been calculated using the DFTB3 and MACE–MP MD simulations, as detailed in a previous work by Purtscher et al.^24^ In both cases a high degree of correlation with the experimental data reported by Koh et al.^11^ is observed. In general, the proposed structure of UMCM-9-β (Figure 2b) shows a more pronounced peak at 2θ = 6.5° compared to UMCM-9-α (Figure 2a), that is not visible in the experimental data. This leads to the conclusion that the experimental structure is indeed UMCM-9-α despite being thermodynamically less favorable.

However, the deviation between the calculated PXRD patterns for the α- and β-form are subtle, and an unambiguous conclusion is not possible based on the available data.

Further analysis on the structural and dynamic properties of UMCM-9-α and -β has been included in the Supporting Information, covering the negative thermal expansion (NTE) behavior (Section S2), and the bulk modulus (Section S3).

### 4.3 Strain Analysis

**Figure 3:**
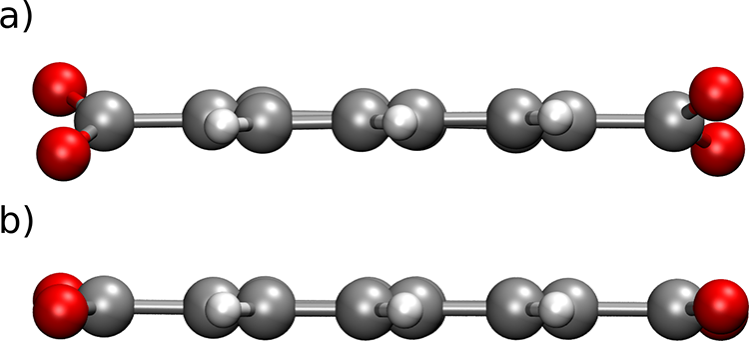
NDC linker in the DFTB optimized structure of (a) UMCM-9-α and (b) UMCM-9-β. The strain of the carboxylate groups in UMCM-9-α is visible, while UMCM-9-β shows a more relaxed structure.

In Figure 3 the torsion angle associated with the carboxylate groups of the NDC linkers in optimized UMCM-9-α (Figure 3a) and -β (Figure 3b) are shown. This serves to highlight the strain in the linkers of UMCM-9-α, which is alleviated in UMCM-9-β due to the adjusted orientation of the SBUs.

Moreover, Figure S3 shows the distribution of the torsion angles between the aromatic moiety and the adjacent carboxylate groups in both BPDC (Figure S3a,c) and NDC (Figure S3b,d) linkers. The distribution has been calculated using the *NPT* trajectories of UMCM-9-α and -β at 298.15 K and 1 atm. It can be seen that the NDC linkers, which are usually found to be coplanar,^71^ exhibit an angle distribution between the aromatic moiety and the adjacent carboxyl groups centered around and angle θ_NDC_ of 25° for UMCM-9-α, in contrast to θ_NDC_ distribution around 10° observed in UMCM-9-β, see Figure S3b. This indicates that the different distribution observed in the NDC linkers is a consequence of the orientation of the SBUs in both UMCM-9 structures. In contrast, the BPDC linkers display a θ_BPDC_ distribution centered around 10° for UMCM-9-α and -β, respectively, indicating that the strain in the BPDC linkers is less pronounced compared to the NDC case, see Figure S3a. This is due to the presence of the biphenyl torsion angle, which can alleviate the strain on the carboxylate groups.

It can be observed, that the MACE–MP model (Figure S3c,d) also introduces a BPDC linker strain in UMCM-9-α, which is not present in the DFTB calculations. This may be attributed to the limitations of the MACE–MP model in accurately representing biphenylic groups, given that the training data is derived from solid-state structures that may not reflect the effect of strained linkers. Overall, this analysis highlights the importance of the strain in the linkers when interpreting the structural properties of MOFs, as this can have a significant impact on the overall stability and properties of the material.^71^ The observed differences in the total energy between the two polymorphs can be directly attributed to the observed strain in the NDC carboxylate groups in case of UMCM-9-α.

A broadening of the distribution in the NDC linkers can be observed with higher temperature, especially in the case of UMCM-9-α (Figure S4a), while it is less pronounced in the β-form (Figure S4b). The distribution in UMCM-9-β is found to be narrower and less affected by temperature compared to UMCM-9-α, which also points toward the fact that the β-form is thermodynamically more stable.

### 4.4 Hydrogen Interaction Energy

The hydrogen adsorption motifs and the interaction energy *U*_int_ have been studied for the 2SBU-NDC model system using geometry optimizations with DFT, as well as DFTB3 and MACE–MP. In addition, geometry optimizations have been performed on the periodic UMCM-9-α and -β with a single H_2_ molecule using DFTB3 and MACE–MP. To study *U*_int_ in a dynamic environment, the *NPT* simulations at 298.15 K and 1 atm have been analyzed using the last 400 ps of the sampled trajectories.

Figure S5 shows the most favorable hydrogen binding motifs of the 2SBU-NDC model system. Since constrained optimizations are not available for DFTB3 and MACE–MP, only the β-form of the model system has been investigated. It can be seen that the hydrogen molecule is located in the vicinity of the Zn_4_O^6+^ cluster in between two Zn tetrahedrons, demonstrating the importance of the partially opened metal sites in the hydrogen adsorption process. Overall, the DFT and DFTB calculations show similar binding motifs, with both hydrogen atoms having similar distances to the central oxide of the cluster. However, MACE–MP fails to reproduce this specific adsorption motif as the hydrogen molecule is rotated by 90° and is coordinated along its bond axis. The *U*_int_ value of these binding motifs for the β-form have been calculated to be −9.8, −9.2, and −7.6 kJ mol^–1^ for DFT, DFTB3, and MACE–MP, respectively. DFT and DFTB3 are in good agreement with a difference below 1 kJ mol^–1^. In contrast, MACE–MP shows both a different binding motif and a lower *U*_int_ value. In case of the α-form *U*_int_ has been found to be −10.8 kJ mol^–1^ for DFT, see Table S6. Although, it should be added that the DFT calculation has been conducted by constraining the torsion angles of the NDC linker, which may have an effect on the interaction energy.

**Figure 4:**
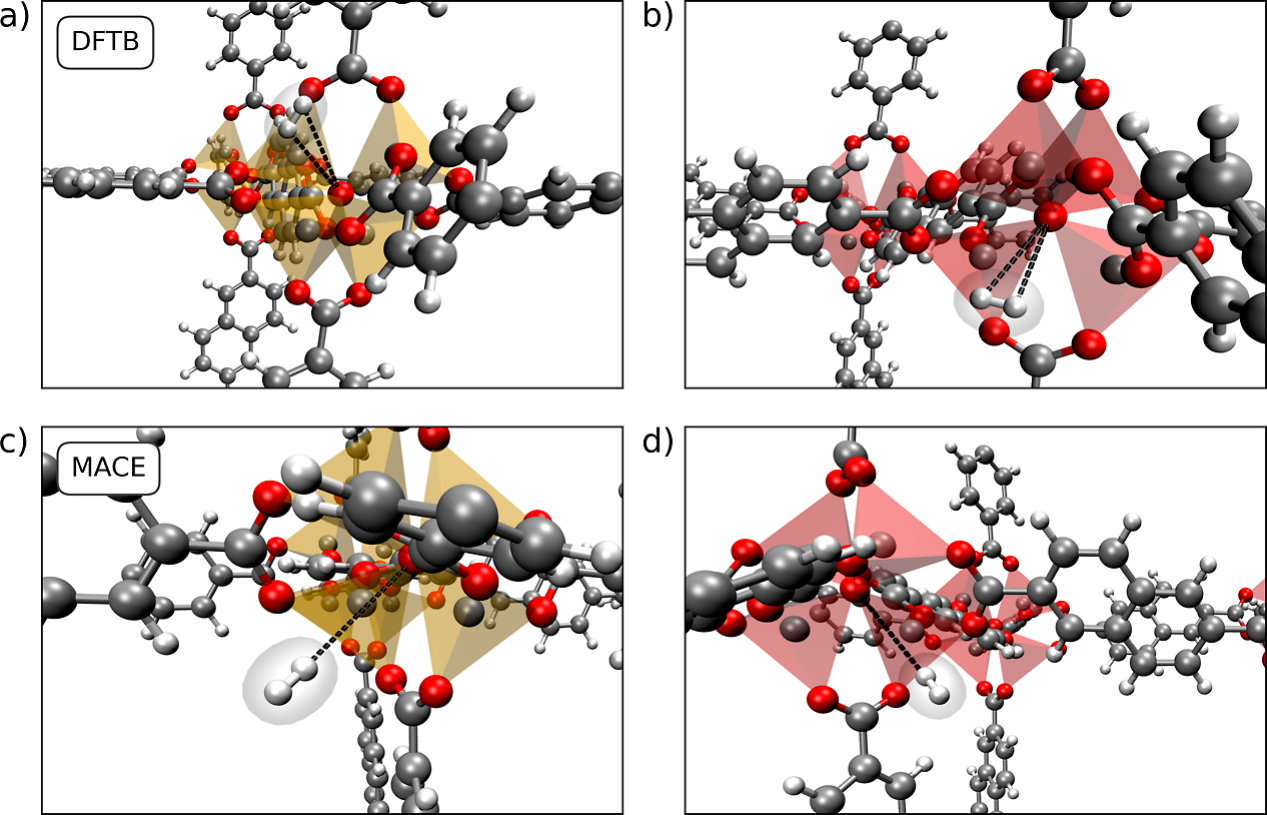
H_2_ interaction motifs within the periodic unit cell of UMCM-9-α (a,c) and UMCM-9-β (b,d) have been analyzed using the DFTB3 and MACE–MP levels of theory.

Furthermore, optimizations of the periodic UMCM-9-α and -β show similar binding motifs, with the hydrogen molecule located near the Zn_4_O^6+^ cluster, see Figure 4. The two different adsorption motifs for DFTB3 and MACE–MP also persist when the periodic unit cell is considered. The *U*_int_ value of the α-form has been found to be −9.5 and −6.8 kJ mol^–1^ for DFTB3 and MACE–MP, see Table S7. The β-form shows a *U*_int_ of −9.9 and −6.8 kJ mol^–1^ for DFTB3 and MACE–MP, respectively. These results are consistent with the 2SBU-NDC model system, where DFT and DFTB3 show similar binding motifs and interaction energies, while MACE–MP displays a weaker binding and a deviating motif.

Figure S6, illustrates the hydrogen binding energy *U*_int_ and its concentration dependence in UMCM-9-α and -β obtained from the DFTB3 simulations. When considering the system with only one H_2_, the *U*_int_ has been found to be −2.0 and −2.1 kJ mol^–1^ for UMCM-9-α and -β, respectively. However, when the number of H_2_ molecules is increased to four, the *U*_int_ value of UMCM-9-α is found to be higher compared to UMCM-9-β yielding −6.9 and −4.4 kJ mol^–1^, respectively. This effect could be attributed to cooperative effects^72^ of the hydrogen atoms in the α-form, which may be less pronounced in the β-form. In addition, the overall higher degree of flexibility of the NDC linkers in the α-form could lead to an increased probability of hydrogen molecules close to the metal nodes, which could result in higher *U*_int_ values. In the following sections the difference in the hydrogen interaction energy will be discussed in the context of H_2_ diffusion in UMCM-9-α and -β, showing that the α-form results in a higher diffusion activation energy and a higher probability of the H_2_ molecules being located in the vicinity of the metal nodes compared to the β-form.

All further analyses of hydrogen storage properties in UMCM-9 will only show the results obtained from DFTB3 simulations, as the MACE–MP model failed to reproduce the correct binding motifs and hydrogen interaction energies.

### 4.5 Hydrogen Diffusion

**Figure 5:**
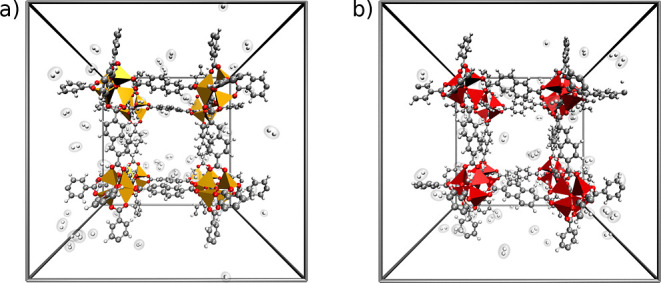
UMCM-9-α (a) and UMCM-9-β (b) containing 64 hydrogen molecules within their respective unit cells.

The hydrogen diffusion properties of UMCM-9-α and -β (Figure 5) have been studied. The simulations provide a comprehensive analysis of the diffusion of hydrogen throughout the porous structure of the MOF material, offering valuable insights into structure and dynamics of hydrogen storage. The unit cells of UMCM-9-α and -β have been loaded with 8, 16, 32, 64, 96, and 104 hydrogen molecules, respectively. The corresponding mole percentages of molecular hydrogen are approximately 0.21, 0.42, 0.83, 1.65, 2.46, and 2.66%, see Section S1 for details. Higher loading levels, such as 112, 120, and 128 H_2_ molecules, have been tested, but the systems were found to be unstable and collapsed during the equilibration phase.

Each system has been equilibrated for 150 ps at the respective temperature and at 1 atm of pressure. The temperature range of 298.15–398.15 K has been selected for the DFTB3 simulations to enable the observation of temperature-dependent diffusion under experimentally accessible conditions. While practical hydrogen storage often operates at cryogenic temperatures^12^ (∼77 K), this higher temperature range has been selected to ensure sufficient molecular mobility for an accurate calculation of self-diffusion coefficients and activation energies.^21^ At lower temperatures the number of diffusive events is diminished on a logarithmic scale, rendering simulations at this range impractical to monitor diffusion via MD simulations. This approach also enables the identification of structural and dynamic differences between the polymorphs within reasonable simulation time, which can offer insights into H_2_ transport mechanisms that persist even at lower temperatures.

The equilibrated systems have then been sampled for 450 ps in order to calculate the self-diffusion coefficients and the associated activation energies of the H_2_@UMCM-9 systems.

**Figure 6:**
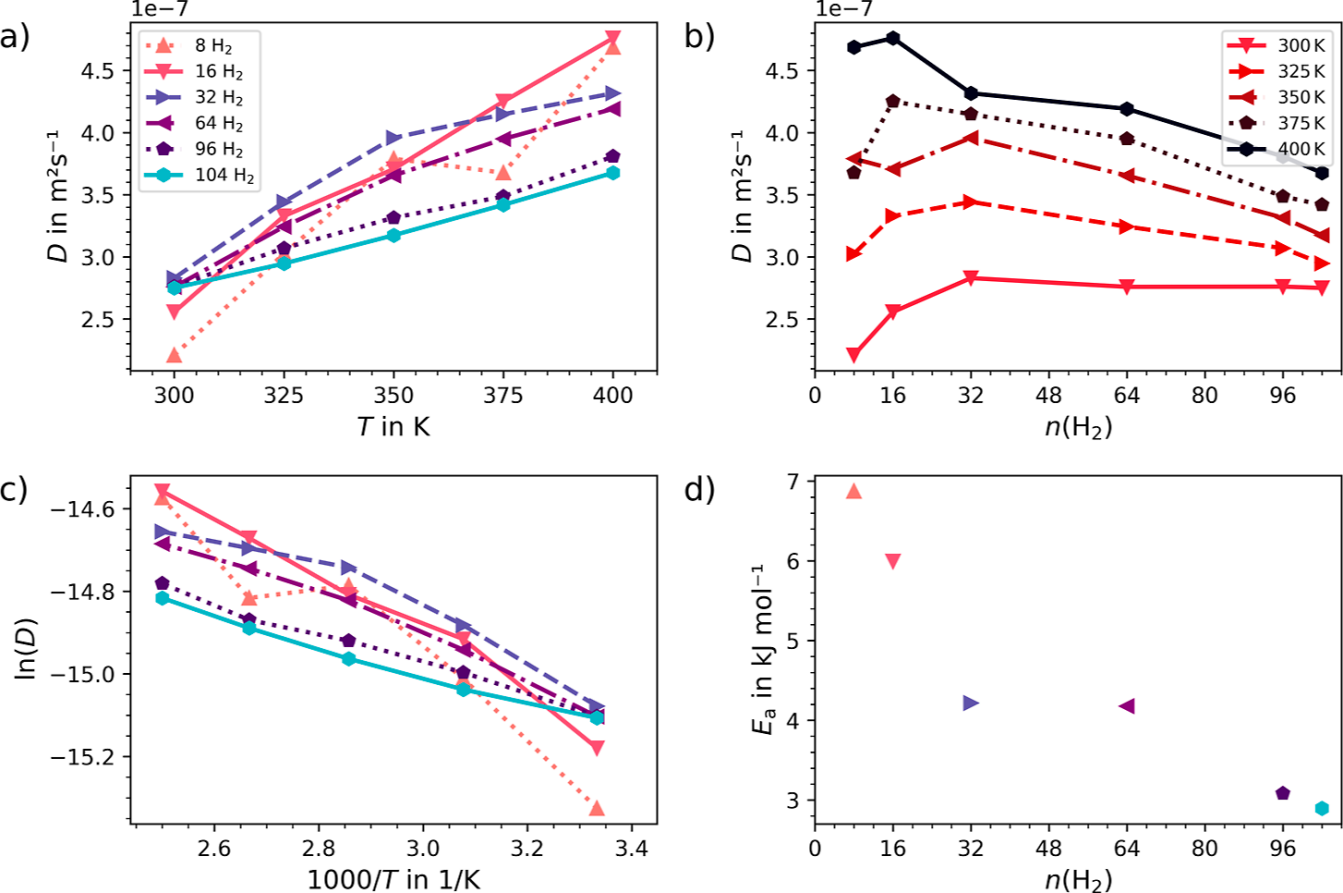
Hydrogen self-diffusion coefficient for (a) varying loadings and (b) temperatures in UMCM-9-α, calculated using the Einstein relation derived from DFTB3 MD simulation trajectories, along with (c) the respective Arrhenius representation of the self-diffusion coefficient and (d) the associated activation energies *E*_a_.

**Figure 7:**
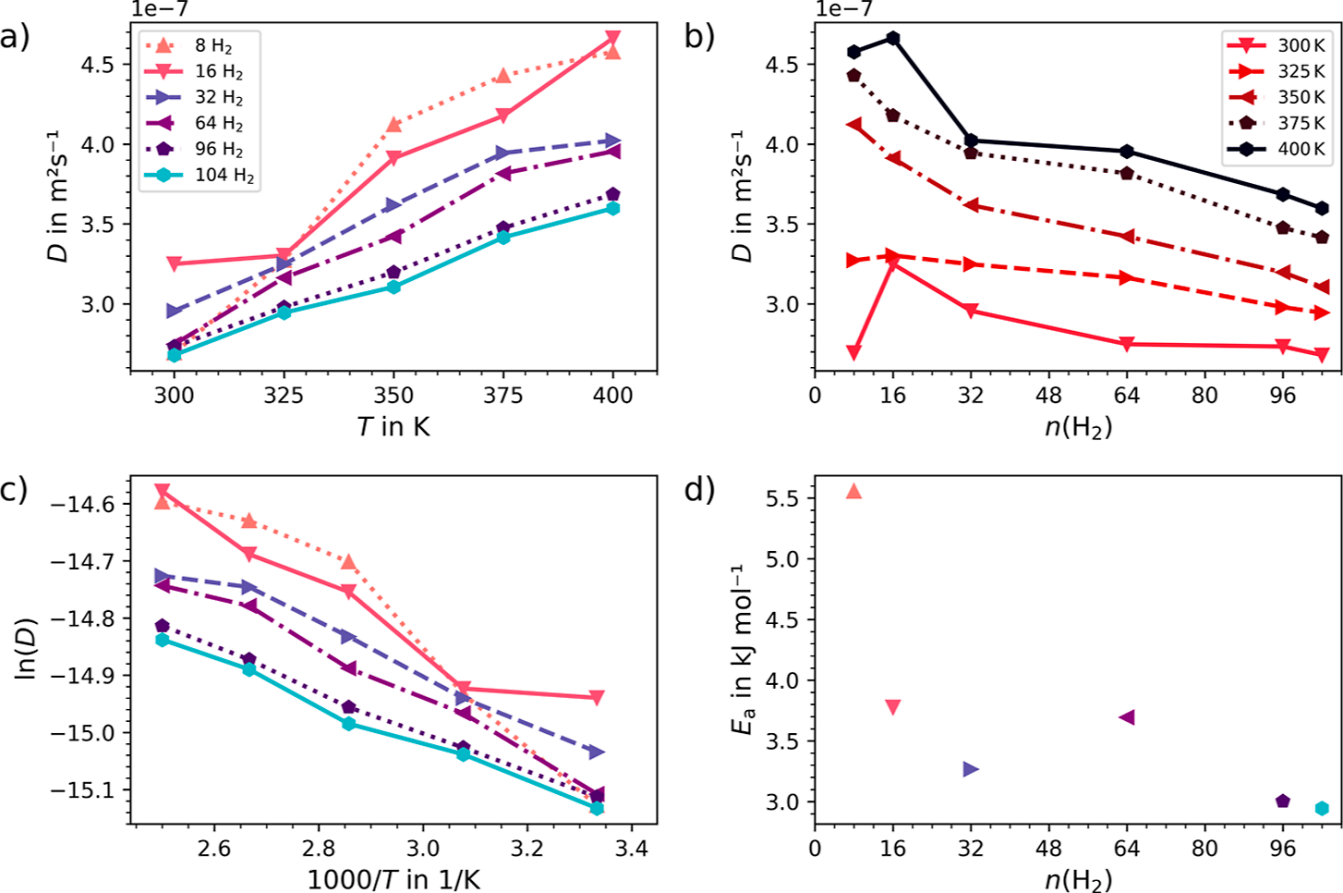
Hydrogen self-diffusion coefficient for (a) varying loadings and (b) temperatures in UMCM-9-β, calculated using the Einstein relation derived from DFTB3 MD simulation trajectories, along with (c) the respective Arrhenius representation of the self-diffusion coefficient and (d) the associated activation energies *E*_a_.

The Einstein relation^73^ has been employed to calculate the hydrogen diffusion coefficients of the sampled systems, as detailed in Section S1. The results are illustrated in Figures 6a–c and 7a–c for UMCM-9-α and -β, respectively. The results demonstrate that the β-form exhibits higher hydrogen self-diffusion compared to the α-form at lower loadings and temperatures. This is attributed to its less strained SBUs, which result in a reduced interaction strength between hydrogen and the framework, seen also by the lower values for *U*_int_ at higher loadings as discussed earlier. This trend is consistent with the activation energies *E*_a_ calculated from the Arrhenius representation of the self-diffusion coefficients, as depicted in Figures 6d and 7d. At higher loadings the *E*_a_ values converge towards 3 kJ mol^–1^, indicating that the structural differences between UMCM-9-α and -β have virtually no impact on the activation energy of hydrogen diffusion. At lower loading levels, the α-form displays a 1.5 kJ mol^–1^ higher *E*_a_ compared to the β-form, which is consistent with the stronger interaction between hydrogen and the framework in UMCM-9-α.

Ultimately, it is evident that the structural differences between the α- and β-form of UMCM-9 directly affect the diffusion of the H_2_ molecules. UMCM-9-α, with its original framework, provides slower hydrogen self-diffusion, which could be beneficial for applications requiring hydrogen storage. By contrast, UMCM-9-β, distinguished by its modified linker orientation and potentially less constrained carboxylate groups, enables slightly enhanced hydrogen mobility.

### 4.6 Hydrogen Radial Distribution

The radial distribution function (RDF) and interaction motifs derived from the DFTB3 MD simulations offer insights into the gas–gas and host–gas interactions. The analyses have been performed on the sampled systems from the DFTB3 diffusion simulations.

**Figure 8:**
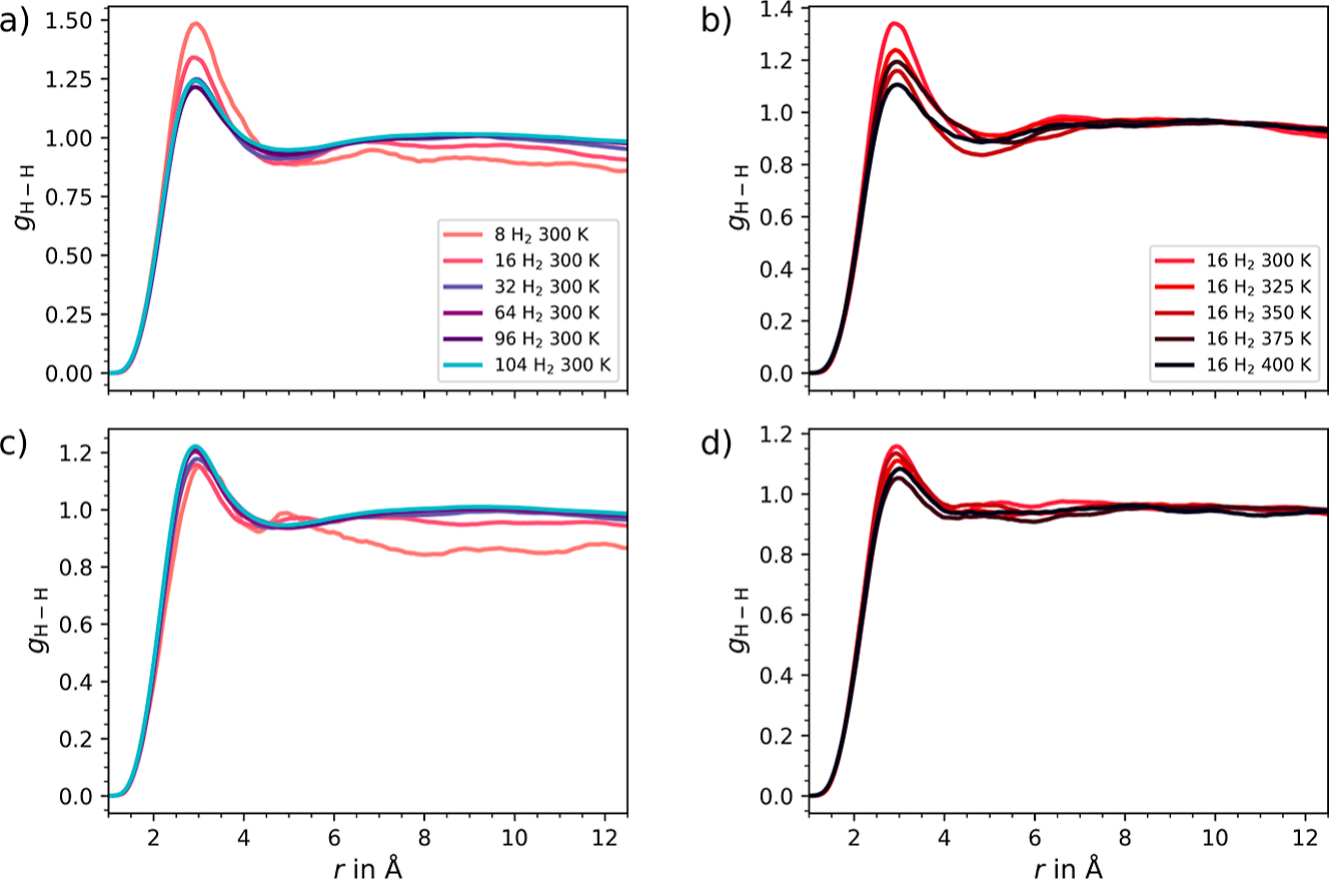
H–H radial distribution calculated of H_2_ molecules in (a,b) UMCM-9-α and (c,d) in UMCM-9-β obtained from the DFTB3 MD simulations. Panels a and c illustrate the influence of varying H_2_ concentrations, whereas panels b and d demonstrate the effect of different temperatures on the distribution of 16 H_2_ molecules.

The distribution function *g*_H–H_ representing the distribution between two H_2_ molecules exhibits comparable behavior in both UMCM-9-α and -β, as illustrated in Figure 8. At elevated concentrations and temperatures, the distribution shows a tendency toward a gas-like behavior, which can be seen in the broader peaks and reduced intensity at 3 Å. At lower concentrations and temperatures, *g*_H–H_ exhibits a more structured distribution and higher ordering after 3 Å. However, in UMCM-9-β the distribution does not seem to be less strongly influenced by the concentration or temperature, as the intensity difference is less pronounced compared to UMCM-9-α.

**Figure 9:**
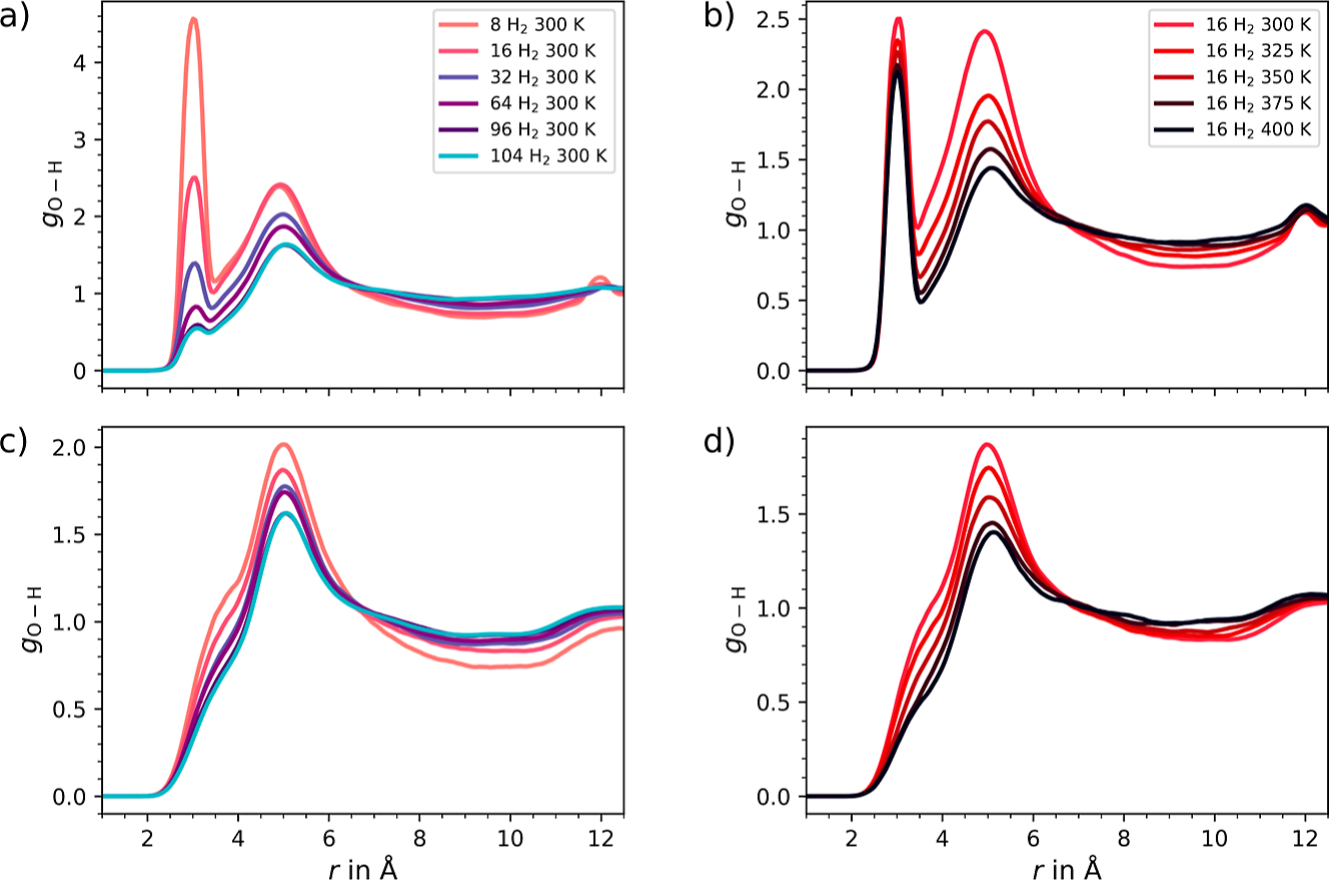
O–H radial distribution calculated of the oxygen in the Zn_4_O^6+^ clusters to H_2_ molecules in (a,b) UMCM-9-α and (c,d) in UMCM-9-β obtained from the DFTB3 MD simulations. Panels a and c illustrate the influence of varying H_2_ concentrations, whereas panels (b,d) demonstrate the effect of different temperatures on the distribution of 16 H_2_ molecules.

The RDF *g*_O–H_, representing the distribution between the oxygen of the Zn_4_O^6+^ clusters and the hydrogen molecules, provides valuable insights into the interaction between the metal nodes and the H_2_ molecules, as shown in Figure 9. UMCM-9-α (Figure 9a,b) and -β (Figure 9c,d) is the intensity and distribution of the RDF peaks in case of the Zn_4_O^6+^ clusters to the hydrogen molecules. Where UMCM-9-α shows a well-defined peak at 3.2 Å and a second broader peak at 5 Å, UMCM-9-β only exhibits a broader peak at 5 Å. This leads to the conclusion that the hydrogen molecules interact more strongly with the Zn_4_O^6+^ clusters in the α-form compared to β-form, which is due to the opening of the strained clusters in UMCM-9-α. Similar to the *g*_H–H_ distribution, the *g*_O–H_ distribution is less influenced by the concentration or temperature in UMCM-9-β compared to UMCM-9-α. Interactions with the metal nodes are known to be crucial for the hydrogen storage capacity of MOFs, as they can significantly influence the adsorption and desorption properties of the material.^74^

### 4.7 Hydrogen Probability Density

**Figure 10:**
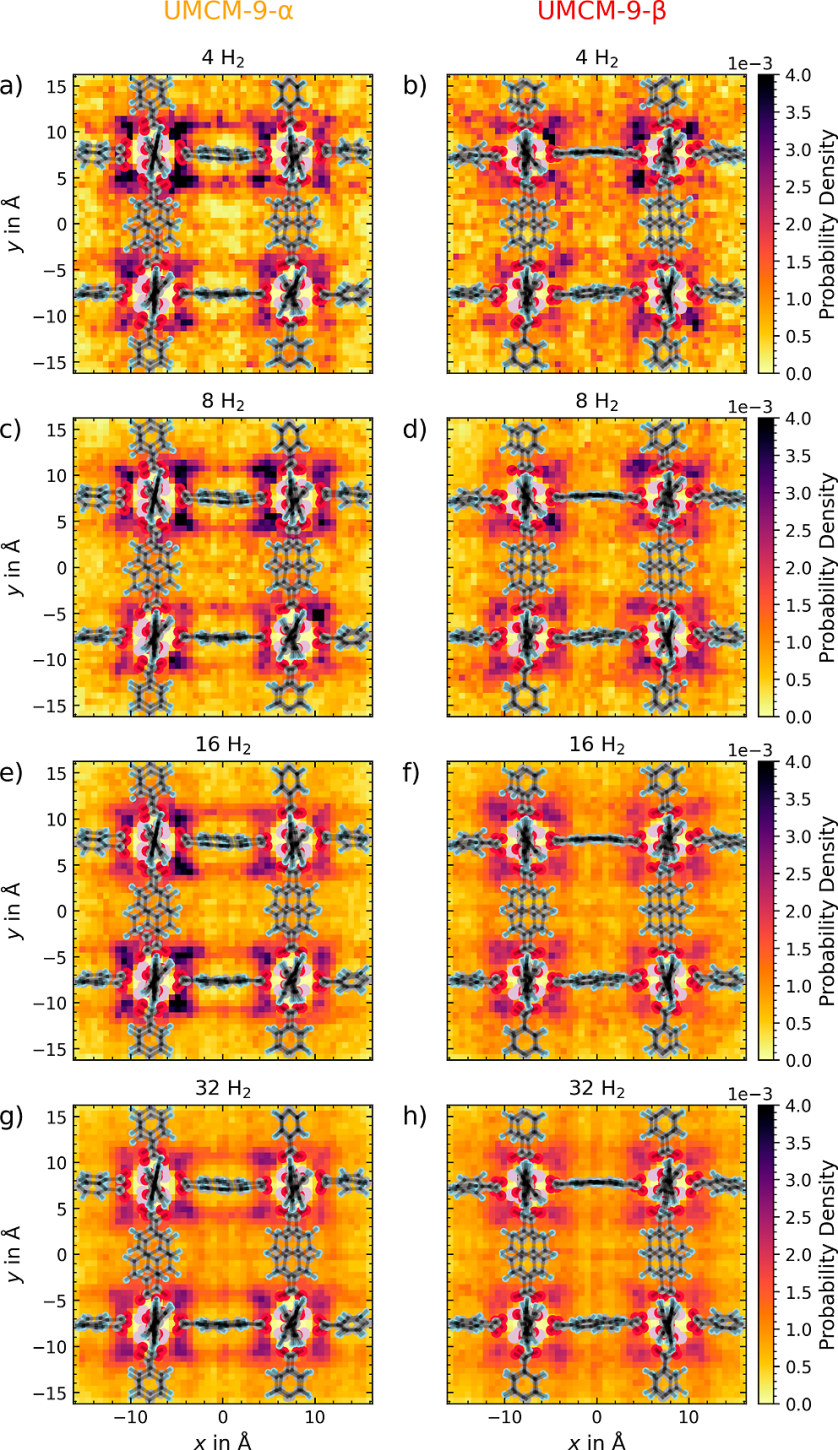
H_2_ density in UMCM-9-α (left) and UMCM-9-β (right) at 298.15 K and 1 atm obtained via DFTB3 MD simulations. The heatmaps show the density of H_2_ molecules in the unit cell at different loadings from 4 to 32 H_2_ molecules.

The hydrogen probability density of UMCM-9-α and -β has been calculated using the DFTB3 simulations. A 2D probability density heatmap, shown in Figure 10, clearly show the previously discussed interactions with the SBUs. The α-polymorph (Figure 10 left) indeed displays a more structured and ordered distribution of H_2_ molecules, while the β-form (Figure 10 right) exhibits a more disordered and less structured distribution. The probability density of H_2_ molecules in UMCM-9-β is more spread out and less localized, indicating a more flexible and dynamic interaction environment. Whereas, UMCM-9-α shows a more structured and ordered probability density of H_2_ molecules, which is in line with the *g*_O–H_ radial distribution. The heatmap also shows that with higher loading the distribution becomes more gas-like, which is expected due to the higher concentration of H_2_ molecules, increasing the probability of collisions between the molecules.

At lower temperatures, the H_2_ molecules are more localized and exhibit a more structured distribution, as shown in Figure S7. Whereas at higher temperatures, the distribution becomes more disordered and less structured, indicating a more gas-like behavior, as shown in Figure S8. This suggests that the temperature has a significant impact on the distribution of H_2_ molecules within the framework, due to the increased kinetic energy of the molecules. At higher temperatures and higher loading of H_2_ molecules lead to a more disordered and less structured distribution of H_2_ molecules, which is in line with the RDF of the H_2_ molecules to the Zn_4_O^6+^ clusters.

This highlights the importance of considering the structural differences between UMCM-9-α and -β when interpreting the interaction motifs and diffusion properties of H_2_ molecules.

## 5 Conclusions

This study provides a detailed investigation into the structural and dynamic properties of two polymorphs of the metal–organic framework UMCM-9 (UMCM-9-α and -β) using molecular simulations, including DFTB3 and MACE–MP. The proposed UMCM-9-β polymorph exhibits reduced strain in the linkers and a higher degree of flexibility in comparison to the α-form, which is characterized by pronounced strain in its NDC linkers. These structural differences are reflected in the material properties, with UMCM-9-β exhibiting a lower bulk modulus and a more negative thermal expansion coefficient. The enhanced flexibility may offer significant advantages in applications where material responsiveness to temperature changes is critical.

The simulations demonstrate that the β-form facilitates faster hydrogen diffusion in comparison to UMCM-9-α. This phenomenon can be attributed to the reduced interaction strength between the hydrogen molecules and the framework in UMCM-9-β. In contrast, the α-form of UMCM-9 exhibits stronger hydrogen-framework interactions, which could be beneficial for applications requiring more stable hydrogen storage, although this may be at the expense of reduced self-diffusion rates. Overall, the interaction with the Zn_4_O^6+^ clusters is more pronounced in UMCM-9-α compared to UMCM-9-β, which is reflected in the hydrogen self-diffusion properties of the two polymorphs. The hydrogen self-diffusion coefficients and activation energies calculated in this study demonstrate the impact of structural variations on gas mobility, thereby emphasizing the potential of UMCM-9-β as a more efficient material for hydrogen storage. Nevertheless, UMCM-9-α may be more suitable for applications where gas retention and slower diffusion are desired.

Alternatively to the 3ob^41,42^ parameter set used for the DFTB3 calculations, the extended matsci parameters set developed by Lukose et al.^23^ could be used to describe Zn-containing MOF systems.

While the MACE–MP potential may not attain the same level of precision as DFTB3, it offers a cost-effective approach for simulating the system’s dynamics and thermodynamic properties. The MACE–MP potential has been trained on a substantial data set of DFT calculations, thereby enabling the prediction of potential energies and forces within the NNP framework. The MACE–MP potential has been demonstrated to be a valuable tool for gaining insights into the MOF’s behavior, including its energetic and structural properties, as well as its thermal and mechanical behavior. It should be noted, however, that the MACE–MP potential is not without limitations in terms of accuracy.

A comparison with DFT and DFTB3 reveals that the MACE–MP potential is unable to accurately predict hydrogen interactions.

Nevertheless, the authors of this work believe that the MACE–MP model may yield a more accurate representation if the model were to be fine-tuned to the systems under investigation.^75^

It is noteworthy that the findings indicate the possibility of directing the synthesis conditions of UMCM-9 to favor the formation of either polymorph, based on thermodynamic or kinetic control and also solvent effects. The thermodynamic product is believed to be UMCM-9-β, which is more stable due to its lower total energy. In contrast, UMCM-9-α is thought to be the kinetic product, forming under conditions that favor faster assembly but result in a higher energy structure. This insight highlights the potential for synthetic strategies to influence the material’s properties by controlling reaction conditions, such as temperature and time. Ultimately, an understanding of the impact of synthesis on these polymorphs could prove crucial for optimizing MOFs in general for specific gas storage or catalytic applications, thereby providing a pathway to tailor material properties through deliberate structural control.

## Data Availability

All calculations have been conducted using the PQ^36^ and PQAnalysis^54^ software packages, which are available at https://github.com/MolarVerse/PQ and https://github.com/MolarVerse/PQAnalysis, respectively. The data that support the findings of this study are available from the corresponding author upon request.

## Supporting Information Available

Simulation protocol details for molecular dynamics simulations, including equilibration and sampling conditions; Analysis of the negative thermal expansion behavior, and bulk modulus of UMCM-9-α and -β; Tables of the optimized potential energy, H_2_ interaction energy and lattice parameters of UMCM-9-α and -β, and the hydrogen interaction energy of the 2SBU-NDC model system; Figures of the linker angle distribution, H_2_ interaction motifs in the 2SBU-NDC model system, and the hydrogen interaction energy of UMCM-9-α and -β calculated from DFTB3 MD simulations; Figures of the H_2_ density in UMCM-9-α and -β at different temperatures and loadings (PDF)
