# Supplementary material for: Comparative Study of UMCM-9 Polymorphs: Structural, Dynamic, and Hydrogen Storage Properties via Atomistic Simulations
Source: J Phys Chem C Nanomater Interfaces. 2025 Mar 4;129(11):5645–55. doi: 10.1021/acs.jpcc.4c07872 (PMC11931535; doi:10.1021/acs.jpcc.4c07872)
Supplement: Supplementary file 1 — jp4c07872_si_001.pdf [file jp4c07872_si_001.pdf]

Supporting Information:

A Comparative Study of UMCM-9 Polymorphs: Structural, Dynamic, and Hydrogen Storage Properties via Atomistic Simulations

Josef M. Gallmetzer, Jakob Gamper, Stefanie Kröll, and Thomas S. Hofer*

Institute of General, Inorganic and Theoretical Chemistry

University of Innsbruck, Innrain 80-82,

6020 Innsbruck, Austria

E-mail: T.Hofer@uibk.ac.at

Phone: +43-512-507-57111. Fax: +43-512-507-57199

S1 Simulation Protocol

The molecular dynamics simulations have been conducted using a two-stage process to ensure thorough equilibration and accurate sampling. All systems have undergone a 150 ps equilibration phase, during which the initial configuration has been relaxed and both temperature and pressure have been stabilized at both the DFTB3 and MACE-MP level of theory. During this equilibration phase, the Nosé-Hoover thermostat[S1–S3] and Berendsen manostat[S4] have been applied in order to maintain constant temperature and pressure conditions. This allows the system to reach a stable equilibrium suitable for the subsequent sampling phase.

After the equilibration, sampling has been performed for 450 ps and 850 ps in case of DFTB3 and MACE-MP, respectively. All sampling simulations have been executed in the NPT ensemble at 1 atm, with the exception of those related to the analysis of the bulk modulus, which have been carried out under NVT conditions at 298.15 K, thereby varying the volume of the simulation cell. All other simulations have been conducted at varying temperatures, ranging from 198.15 K to 398.15 K in 25 K intervals, in order to investigate the temperature dependence of hydrogen diffusion and its interaction with UMCM-9. Hydrogen gas molecules have been introduced at varying loadings, with a range of 1 to 128 H_2 molecules. The corresponding weight percentages of H_2 loadings in UMCM-9 are provided in Table S1. Higher loading levels, such as 112, 120, and 128 H_2 molecules, have been tested, but the systems were found to be unstable and collapsed during the equilibration phase.

Table S1: Weight percentage of simulated H_2 loadings in UMCM-9. The higher loadings of 112, 120, and 128 H_2 molecules were found to be unstable and collapsed during the equilibration phase.

n(H_2)	wt%
1	0.03
2	0.05
4	0.11
8	0.21
16	0.42
32	0.83
64	1.65
96	2.46
104	2.66
112	2.86
120	3.06
128	3.25

The data obtained from both the MACE-MP and DFTB3 simulations have been subsequently subjected to comprehensive analysis in order to derive insights into the structural and dynamic properties of the system.

In order to analyze the hydrogen diffusion, the mean square displacement (MSD) has been calculated using a correlation window of 20 ps (5000 MD configurations). This approach was employed in order to ensure a reliable estimation of the diffusion coefficient. To calculate the diffusion coefficients, a linear fit to the last 10 ps of the MSD has been performed and the self-diffusion coefficient D has been calculated using the Einstein relation. The associated activation energy E_a has been determined using the Arrhenius equation.

S2 Thermal Expansion

The thermal expansion properties of the α- and β-form of UMCM-9 have been investigated from MD simulations at 5 different temperatures in the range from 248.15 to 348.15 K at DFTB and MACE-MP level of theory. The thermal expansion coefficients α have been calculated using a 5-point stencil derivation, which is provided in more detail in a previous work.[S5]

Since the similarity between the linkers of UMCM-9 and MOF-5, it is expected that both systems exhibit a similar thermal expansion. Analogous investigations employing DFTB3 conducted for MOF-5[S5,S6] and ZIF-8[S6] proved to be in very good agreement with experimental data. Therefore, similar performance can be expected for UMCM-9.

Both α- and β-form of UMCM-9 exhibit a negative thermal expansion coefficient, which is a common feature in MOFs and is attributed to the flexibility of the linkers in the framework.[S7,S8] The DFTB3 simulations yielded thermal expansion coefficients of -18.3 MK^{-1} and -60.1 MK^{-1} for UMCM-9-α and -β, respectively. In contrast, the MACE-MP model yielded more negative coefficients, being -78.5 MK^{-1} and -160.7 MK^{-1}, respectively, see Tab. S2.

The lower thermal expansion of UMCM-9-β in comparison to UMCM-9-α is likely attributed to the decreased flexibility resulting from the higher strain observed in the linkers of the α-polymorph.

Table S2: Thermal expansion coefficients of UMCM-9-α and -β obtained *via* DFTB3 and MACE-MP MD simulations, respectively. These values have been calculated using a 5-point stencil derivation, as outlined in a previous work.[S5] The molecular mechanics (MM) values have been reported by Wieme *et al.*[S9]

	α_0 / MK^{-1}	
	UMCM-9-α	UMCM-9-β
DFTB3	-18.3	-60.1
MACE-MP	-78.5	-160.7
MM[S9]	-37.5	-

As illustrated in Figs. S1 and S2, the lattice parameters a and V of UMCM-9-α and

-β have been plotted as a function of temperature. It is apparent that in case of DFTB3 simulations both structures seem to more strongly deviate from a linear thermal expansion behavior, compared to the MACE-MP. This difference could be attributed to the lower sampling time of the DFTB3 (450 ps) simulations, which might not be sufficient to capture the full thermal behavior of the system, compared to the MACE-MP simulations (850 ps). This highlights the importance of considering the limitations of simulation methods when interpreting results and emphasizes the need for complementary approaches to validate findings. Nevertheless, the DFTB3 simulation results are closer to molecular mechanics (MM) MD literature values of -37.5 MK^{-1} for UMCM-9-α, where Wieme *et al.*[S9] fitted the MD ensemble data using a linear polynomial.

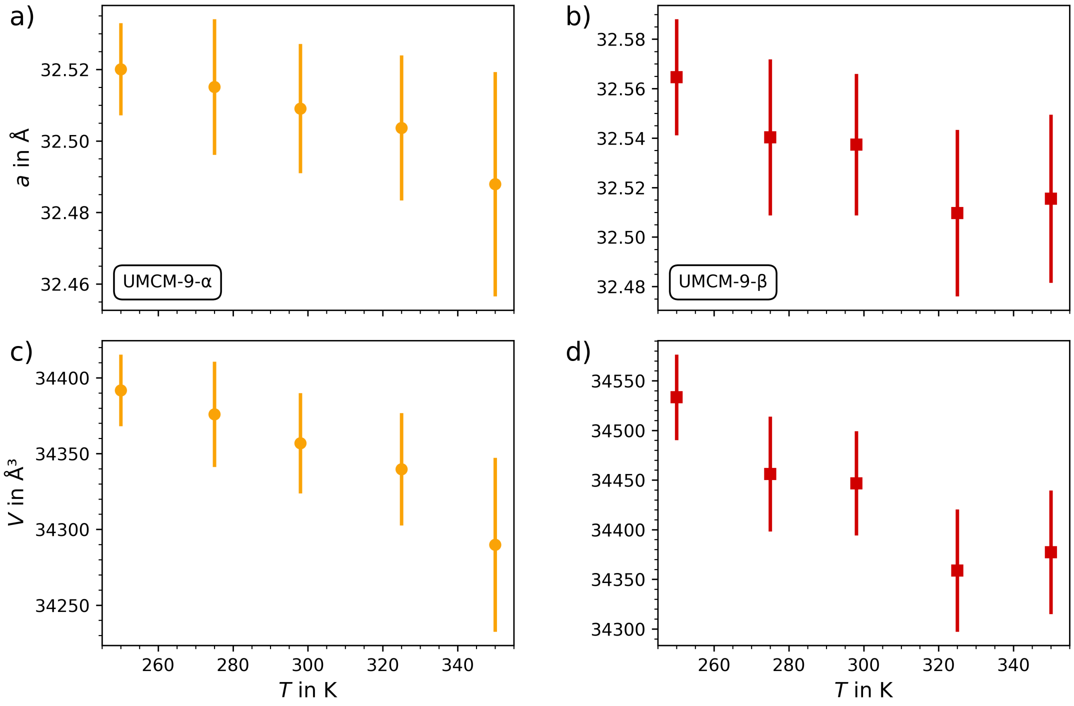

Figure S1: The negative thermal expansion of UMCM-9-α (a, c) and UMCM-9-β (b, d) obtained *via* DFTB3 MD simulations. In panels a) and b), the lattice parameter a is plotted as a function of temperature, while panels c) and d) show the volume V as a function of temperature.

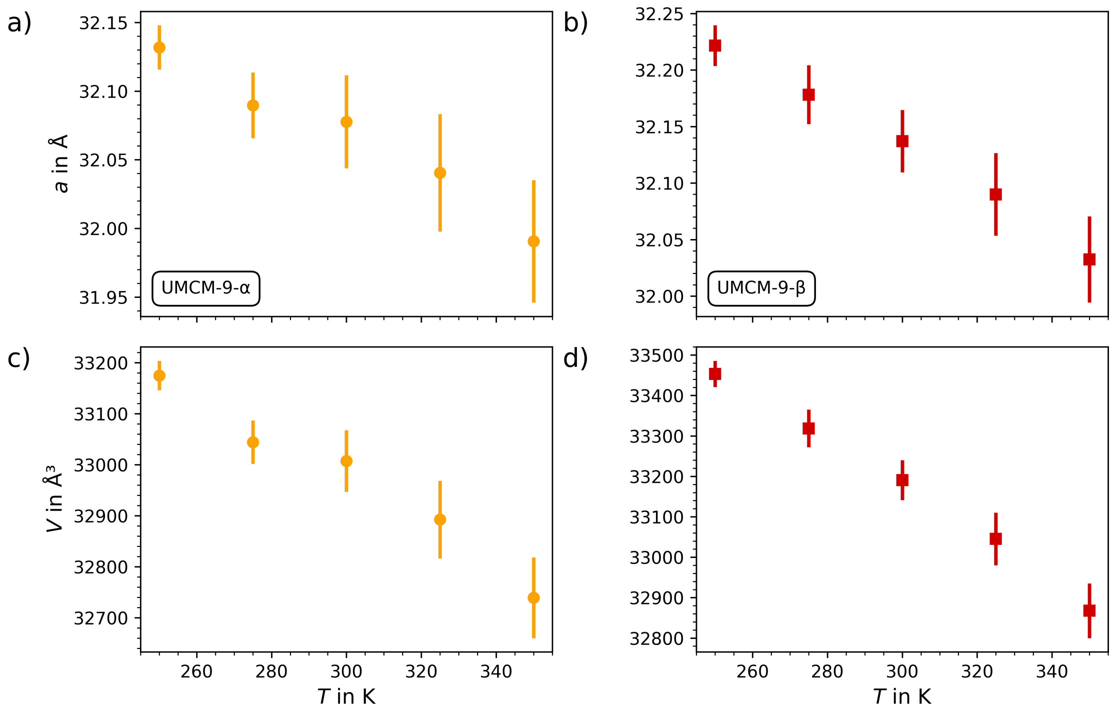

Figure S2: Thermal expansion of UMCM-9-α and -β with MACE-MP. Panels a) and b) show the lattice parameters a change of UMCM-9-α and -β, respectively. Panels c) and d) show the temperature dependence of volume change.

S3 Bulk Modulus

The bulk modulus investigations of UMCM-9-α and -β have been performed using NVT simulations of the equilibrated volumes at 298.15 K and 1 atm, as detailed in Tab. S3. The bulk modulus β has been calculated using a central-difference derivation with a $\pm 0.1\%$ change in volume, see previous work.[S5] The results indicate that UMCM-9-β exhibits a slightly lower bulk modulus of 4.13 GPa compared to UMCM-9-α 4.26 GPa, which is consistent with the increased flexibility[S10] and thermal expansion observed in UMCM-9-β when using DFTB3 simulations. The values reported by Wieme *et al.*[S9] are in good agreement with those obtained using DFTB3 simulations, with a bulk modulus of 4.7 GPa for UMCM-9. While the bulk modulus of both systems has been found to be quite similar, with a difference of 0.13 GPa between UMCM-9-α and -β using DFTB3 simulations.

Alternatively, the MACE-MP simulations indicate a difference of 0.05 GPa between UMCM-9-α and -β with a bulk modulus of 2.71 GPa and 2.76 GPa, respectively. In contrast, MACE-MP simulations yield a bulk modulus that is approximately 57 % of the value reported by Wieme *et al.*[S9]

Table S3: Bulk modulus of UMCM-9-α and -β obtained *via* DFTB3 and MACE-MP MD simulations, respectively. These values have been calculated using a central-difference derivation with a 0.1% change in volume, as outlined in a previous work.[S5] The molecular mechanics (MM) values have been reported by Wieme *et al.*[S9]

	β_0 / GPa	
	UMCM-9-α	UMCM-9-β
DFTB3	4.26	4.13
MACE-MP	2.71	2.76
MM[S9]	4.7	-

Although the longer simulation time of the MACE-MP simulations may provide a more accurate representation of the thermal and mechanical behavior of the system, the DFTB3 simulations yield results that are in closer agreement with the literature values.

S4 Tables

Table S4: Potential energy difference ΔE obtained from the structure optimizations and MD simulations (298 K and 1 atm) of UMCM-9-α and -β using DFTB3 and MACE-MP, respectively. Additionally, the energy difference of the non-periodic 2SBU-NDC model system has been calculated using PBE, both unconstrained and constrained.

		ΔE / kJ mol^{-1}
OPT	DFTB	106.1
	MACE-MP	77.2
MD	DFTB	125.9
	MACE-MP	130.9
2SBU-NDC	PBE (unconst.)	$0.3 \cdot 10^{-3}$
	PBE (const.)	15.5

Table S5: Lattice parameter a of the α- and β-form of UMCM-9 at DFTB3 and MACE-MP level of theory compared to the experimental value from Koh *et al.*[S11] The difference Δa is calculated as the difference between the α- and β-form. The lattice parameters have been derived from geometry optimizations and from MD simulations at 298.15 K and 1 atm.

		a(α) / Å	a(β) / Å	Δa / Å
OPT	DFTB3	32.528	32.625	0.097
	MACE-MP	32.306	32.467	0.161
MD	DFTB3	32.509	32.537	0.028
	MACE-MP	32.078	32.137	0.059
Exp.[S11]		32.5	-	-

Table S6: Interaction energy U_{int} of H_2 and UMCM-9-α and -β obtained from the geometry optimized non-periodic model system containing 2 Zn_4O^{6+} clusters capped with formate groups and connected by a single NDC linker (2SBU-NDC), similar to the system used by Schmid *et al.*[S12] The interaction energy has been calculated using the PBE level of theory.

	$U_{\text{int}}(\alpha)$ / kJ mol^{-1}	$U_{\text{int}}(\beta)$ / kJ mol^{-1}
PBE	−10.8	−9.8
DFTB3	–	−9.2
MACE-MP	–	−7.6

Table S7: Interaction energy U_{int} of H_2 and UMCM-9-α and -β for the respective periodic unit cells calculated using the energy minimized configurations obtained at DFTB3 and MACE-MP level of theory, respectively.

	$U_{\text{int}}(\alpha)$ / kJ mol^{-1}	$U_{\text{int}}(\beta)$ / kJ mol^{-1}
DFTB3	-9.5	-9.9
MACE-MP	-6.8	-6.8

S5 Figures

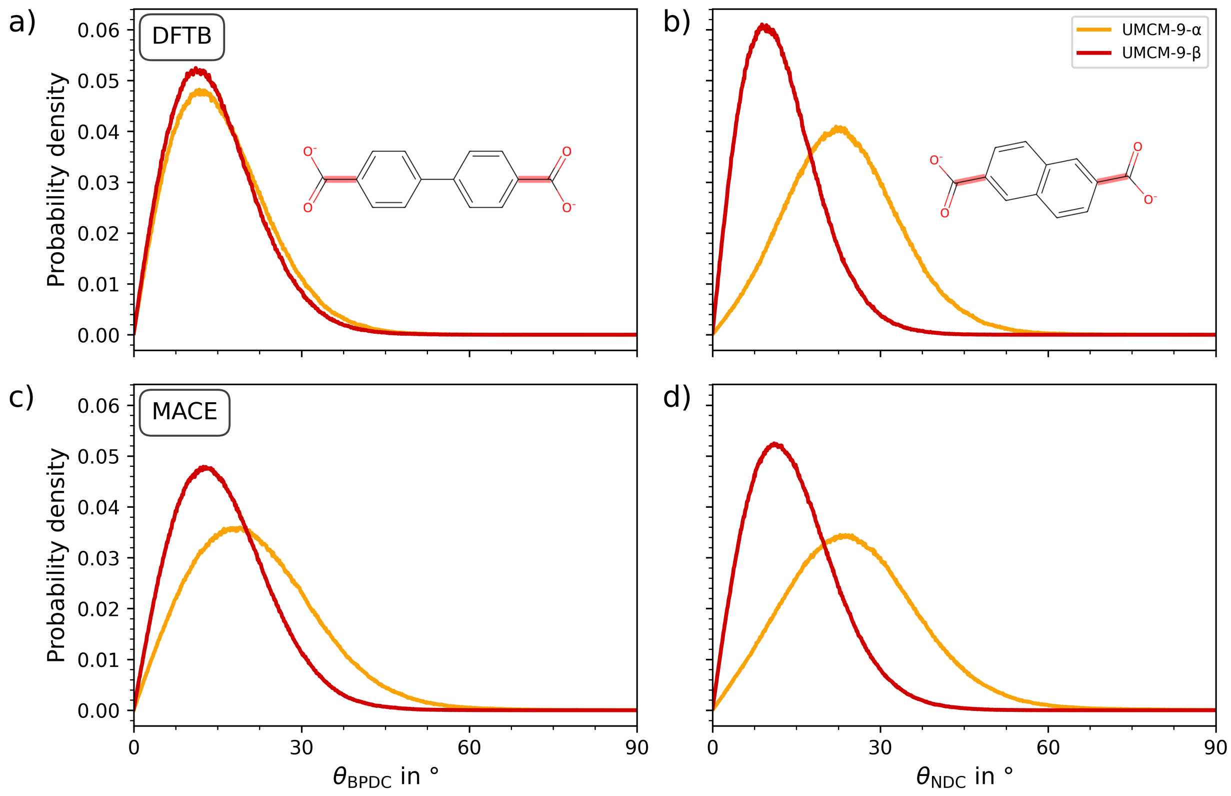

Figure S3: Probability density of the dihedral angles between the aromatic moiety and the adjacent carboxyl group in both BPDC and NDC linkers. The different torsion angles are associated to strain in (a, c) BPDC and (b, d) NDC obtained from MD simulations at (a, b) DFTB and (c, d) MACE-MP level, respectively.

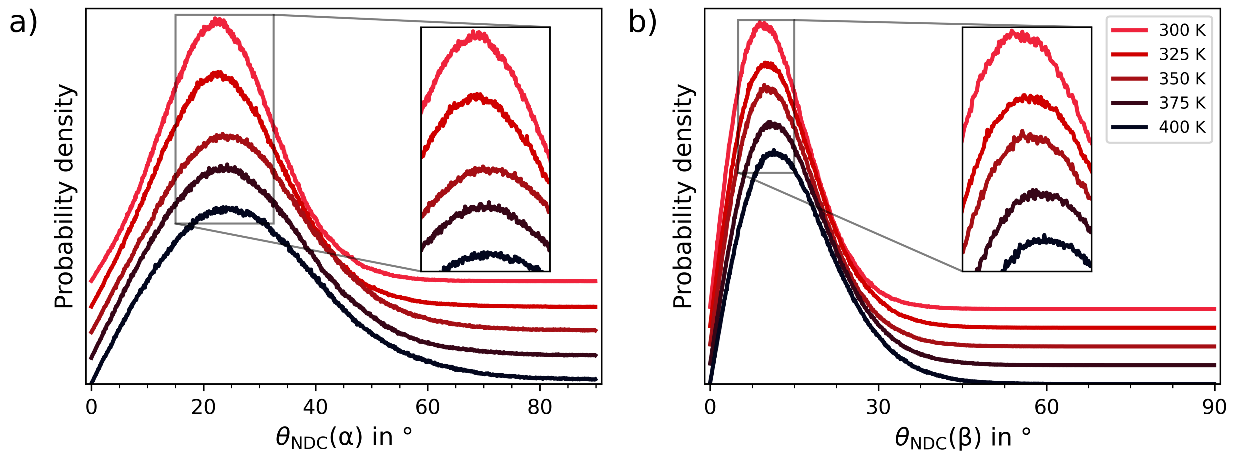

Figure S4: Temperature dependence of the probability density associated with the dihedral angle between the aromatic moiety and the adjacent carboxylate group of NDC. UMCM-9-α (a) and -β (b) probability densities have been obtained from the DFTB3 MD simulations.

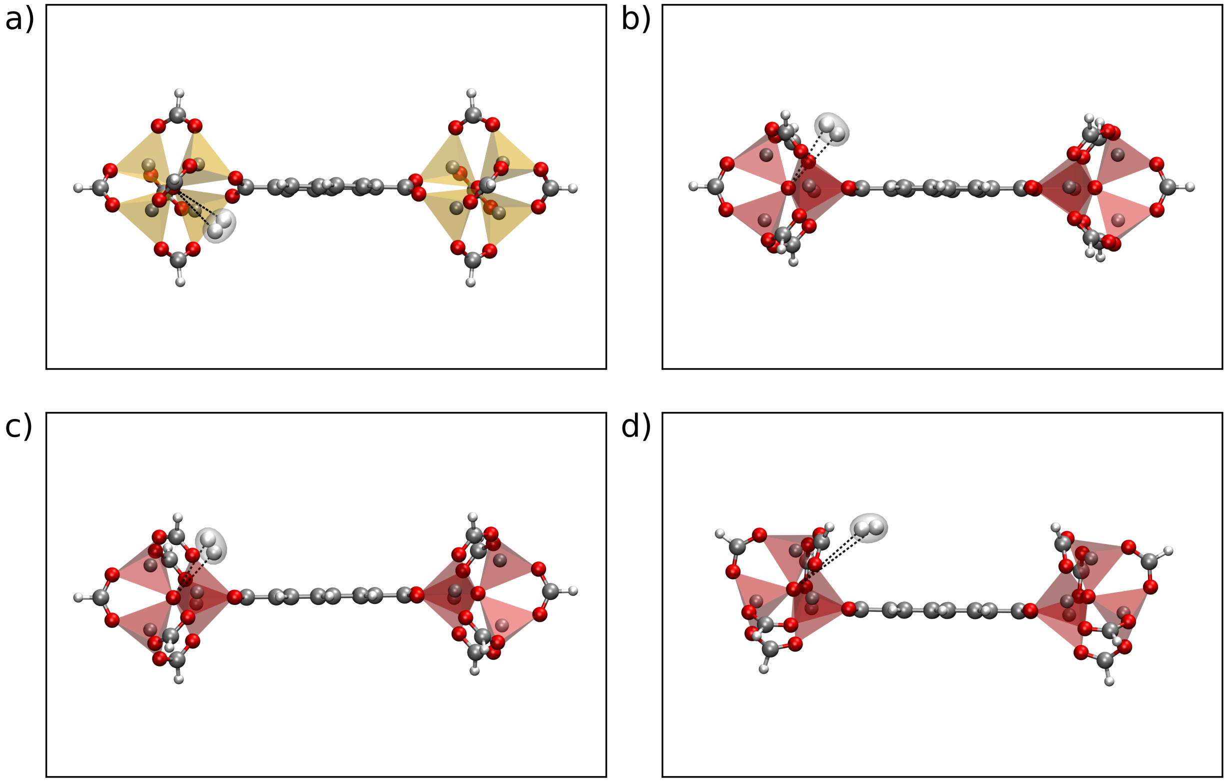

Figure S5: H_2 interaction motifs within the 2SBU-NDC model system have been analyzed using various computational methods, being the (a, b) PBE, (c) DFTB3, and (d) MACE-MP levels of theory, respectively. (a) represents the constrained α-form of UMCM-9, while (b), (c), and (d) correspond to the unconstrained β-form.

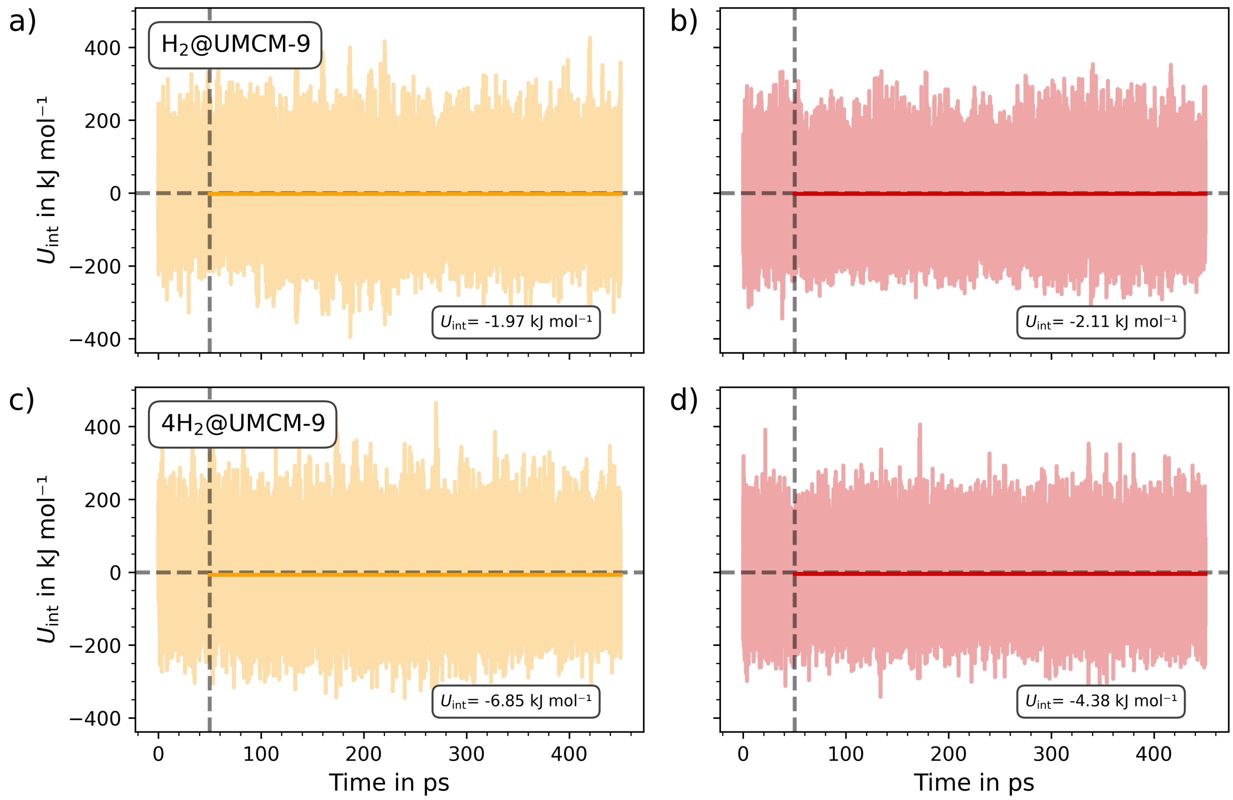

Figure S6: The interaction energy U_{int} in (a, c) UMCM-9-α and (b, d) UMCM-9-β with a loading of (a,b) one and (c,d) four H_2 molecules calculated from the respective DFTB3 MD simulations.

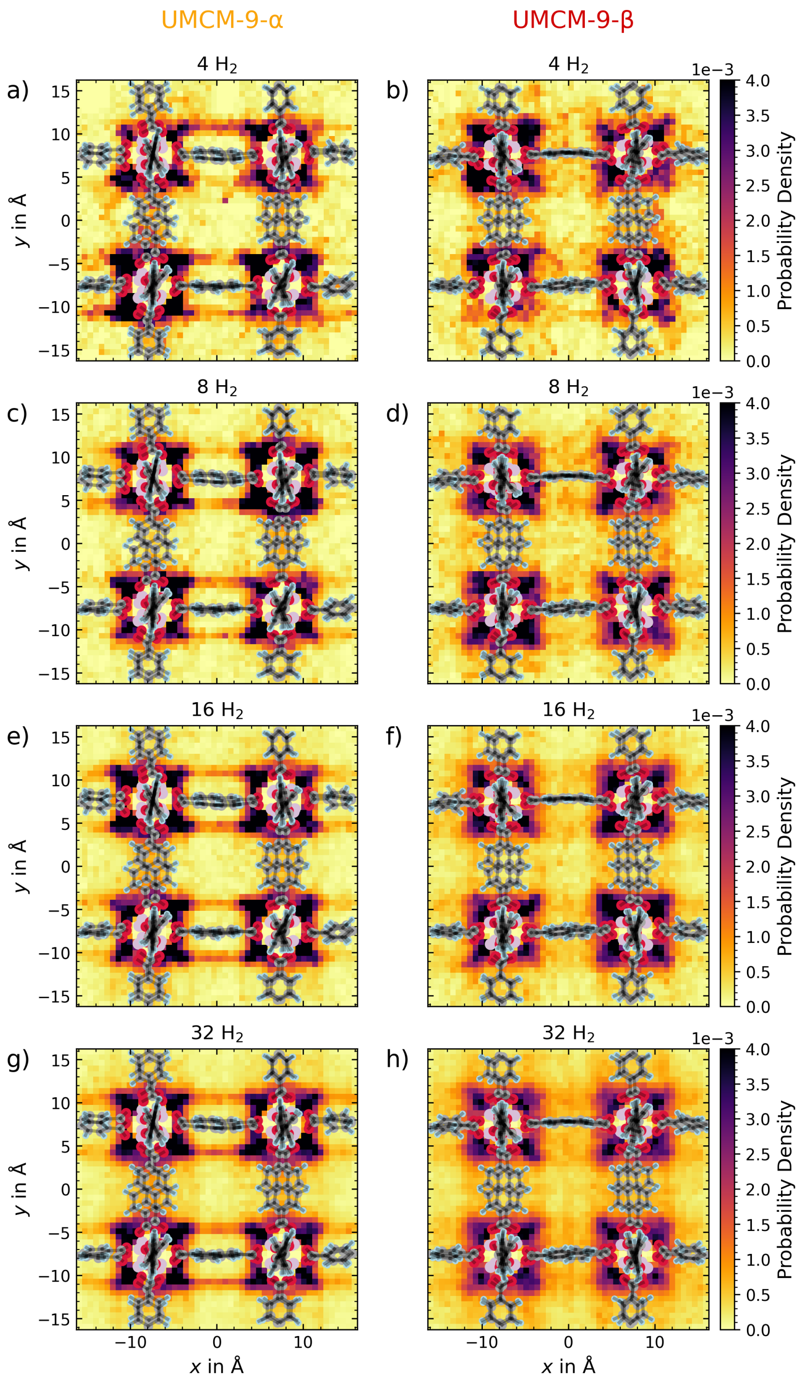

Figure S7: H_2 density in UMCM-9-α (left) and UMCM-9-β (right) at 198.15 K and 1 atm obtained *via* DFTB3 MD simulations. The heatmaps show the density of H_2 molecules in the unit cell at different loadings from 4 to 32 H_2 molecules.

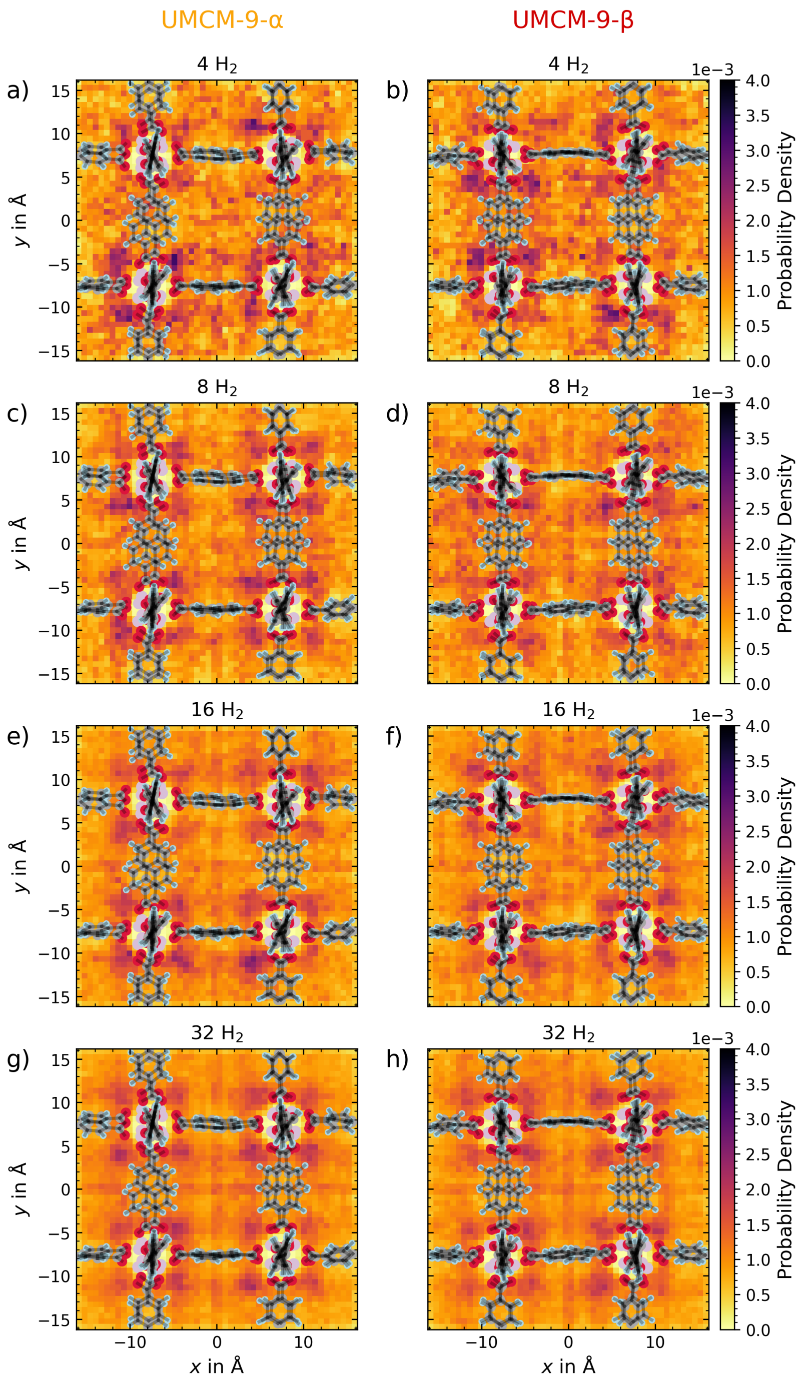

Figure S8: H_2 density in UMCM-9-α (left) and UMCM-9-β (right) at 398.15 K and 1 atm obtained *via* DFTB3 MD simulations. The heatmaps show the density of H_2 molecules in the unit cell at different loadings from 4 to 32 H_2 molecules.

References

(S1) Nosé, S. A unified formulation of the constant temperature molecular dynamics methods. *The Journal of Chemical Physics* **1984**, *81*, 511–519.

(S2) Hoover, W. G. Canonical dynamics: Equilibrium phase-space distributions. *Phys. Rev. A* **1985**, *31*, 1695–1697.

(S3) Evans, D. J.; Holian, B. L. The Nose–Hoover thermostat. *The Journal of Chemical Physics* **1985**, *83*, 4069–4074.

(S4) Berendsen, H. J. C.; Postma, J. P. M.; van Gunsteren, W. F.; DiNola, A.; Haak, J. R. Molecular dynamics with coupling to an external bath. *The Journal of Chemical Physics* **1984**, *81*, 3684–3690.

(S5) Listyarini, R. V.; Gamper, J.; Hofer, T. S. Storage and diffusion of carbon dioxide in the metal organic framework MOF-5 – A semi-empirical molecular dynamics study. *J. Phys. Chem. B* **2023**, *127*, 9378–9389.

(S6) Purtscher, F. R. S.; Christanell, L.; Schulte, M.; Seiwald, S.; Rödl, M.; Ober, I.; Maruschka, L. K.; Khoder, H.; Schwartz, H. A.; Bendeif, E.-E.; Hofer, T. S. Structural properties of metal-organic frameworks at elevated thermal conditions via a combined density functional tight binding molecular dynamics (DFTB MD) approach. *J. Phys. Chem. C Nanomater. Interfaces* **2023**, *127*, 1560–1575.

(S7) Evans, J. D.; Dürholt, J. P.; Kaskel, S.; Schmid, R. Assessing negative thermal expansion in mesoporous metal–organic frameworks by molecular simulation. *J. Mater. Chem. A Mater. Energy Sustain.* **2019**, *7*, 24019–24026.

(S8) Balestra, S. R. G.; Bueno-Perez, R.; Hamad, S.; Dubbeldam, D.; Ruiz-Salvador, A. R.; Calero, S. Controlling thermal expansion: A metal–organic frameworks route. *Chem. Mater.* **2016**, *28*, 8296–8304.

(S9) Wieme, J.; Van Speybroeck, V. Unravelling thermal stress due to thermal expansion mismatch in metal–organic frameworks for methane storage. *J. Mater. Chem. A Mater. Energy Sustain.* **2021**, *9*, 4898–4906.

(S10) Redfern, L. R.; Farha, O. K. Mechanical properties of metal-organic frameworks. *Chem. Sci.* **2019**, *10*, 10666–10679.

(S11) Koh, K.; Van Oosterhout, J. D.; Roy, S.; Wong-Foy, A. G.; Matzger, A. J. Exceptional surface area from coordination copolymers derived from two linear linkers of differing lengths. *Chem. Sci.* **2012**, *3*, 2429.

(S12) Amirjalayer, S.; Schmid, R. Conformational Isomerism in the Isoreticular Metal Organic Framework Family: A Force Field Investigation. *The Journal of Physical Chemistry C* **2008**, *112*, 14980–14987.
